# Contemplating Help-Seeking in Perinatal Psychological Distress—A Meta-Ethnography

**DOI:** 10.3390/ijerph18105226

**Published:** 2021-05-14

**Authors:** Minna Anneli Sorsa, Jari Kylmä, Terese Elisabet Bondas

**Affiliations:** 1 Child Psychiatry, Tampere University Hospital, 33521 Tampere, Finland; 2 Faculty of Social Sciences, Nursing Science, Tampere University, 33520 Tampere, Finland; jari.kylma@tuni.fi; 3 Faculty of Medicine, Nursing Science, University of Oulu, 90014 Oulu, Finland; 4 Faculty of Pharmacy, University of Helsinki, 00014 Helsinki, Finland; 5 Faculty of Health Sciences, University of Stavanger, 4036 Stavanger, Norway; terese.e.bondas@uis.no

**Keywords:** help-seeking behavior, meta-ethnography, meta-synthesis, perinatal mental health, perinatal depression/anxiety, prevention, treatment

## Abstract

Perinatal psychological distress (PPD) may cause delays in help-seeking in the perinatal period, which is crucial for families with small children. Help-seeking theories focus on rational processes of behavior wherein ‘help-seeking’ is viewed as a decision-making process, in which action is preceded by recognizing a problem. We identified the phase prior to actual help-seeking actions as a life situation and a phenomenon through which to gain a deeper understanding from women’s own perspectives. The aim of this study was to integrate and synthesize knowledge of women’s experiences of contemplating seeking help for PPD. We chose interpretative meta-ethnography by Noblit and Hare (1988) and implemented eMERGe guidelines in reporting. The search was performed systematically, and the 14 included studies were evaluated with Critical Appraisal Skills Programme checklist (CASP). We identified seven themes and a metaphor in a lines-of-argument synthesis, showing that contemplating help-seeking is a multidimensional phenomenon. We did not observe a straightforward and linear process (as previous research suggests) but instead a complex process of contemplating help-seeking. A clinical implication is that service providers should work with outreach and develop their tools to connect with mothers with PPD. Another suggestion is to improve training in mental health literacy prior to or during pregnancy.

## 1. Introduction

Experiences causing suffering for women during the perinatal period (pregnancy and the year following childbirth) are formed by culturally constructed encounters. Help-seeking is not solely related to diagnostic questions [1]. Therefore, we chose to focus on perinatal psychological distress (PPD), which has been identified as a holistic emotional state of discomfort that is experienced by an individual in response to specific stressors [2]. Psychological distress involves depression, stress, and anxiety [3]. PPD may be reflected in a person’s behavior, such as adversely affecting interpersonal relationships. It is not well-defined, but it is closely linked to stress and coping [4]. Women may be unable to recognize their symptoms and name them as, e.g., depression [5]. PPD is associated with a number of poor outcomes for the mother, baby, and family, since PPD has an impact on parenting capacities, which is why also infants are at risk of adverse childhood experiences [6,7,8].

Psychological distress during the perinatal period is common, with rates at 21.2% prenatally and 26.7% postnatally [3]. Women are at higher risk for distress if they have low social support, are single, are less educated, are unemployed or experiencing financial instability, are older, use tobacco or alcohol, or have prior psychological problems [9]. In a French nationally representative cohort study, 12.6% of pregnant women reported prenatal psychological distress [10]. In a US national study, the prevalence of serious psychological distress in the previous month ranged from 3.9 to 6.4% during pregnancy and 4.6 to 6.9% postnatally [9]. The prevalence of perinatal depression is 11.4% in high-income countries [11]. Of those who screened positive for perinatal depression, 23% suffered from bipolar disorder [12].

The most widely used framework in help-seeking research is the theory of planned behavior [13,14]. Help-seeking viewed as a decision-making process emphasizes the assumption that persons are active and goal-directed and that they fulfill instrumental purposes by seeking help from somebody, such as for specific mental health concerns [13,14,15]. At an individual level, situational aspects and the person’s background influence their help-seeking behavior and abilities by activating their beliefs about help-seeking; persons also weigh the benefits and utility in relation to the risks of their action [13]. When a person contemplates whether help-seeking is necessary, they decide whether change is necessary [16]. If a person decides that change is needed, she first tries to effect change through her own means, with self-help efforts, before making the decision to seek help from social and health care [16]. Help-seeking is a person-centered phenomenon incorporating the help-seeker’s perspective, which differs from the supply side (defined in this context as access to care).

Saint Arnault [1] has explained that help-seeking is connected to social determinants and cultural humility, as people’s perspectives, values, and preferences need to be taken into consideration in help-seeking encounters. Women’s inability to disclose their feelings can be exacerbated by family members’ and health professionals’ reluctance to respond to mothers’ emotional and practical needs [5]. Many women try and fail to live up to the idealized societal depiction of motherhood; however, each mother is unique, and thus the motherhood experience is different for all mothers [17].

It is important to study help-seeking in the context of perinatal mental health, since PPD often goes undetected and women do not use the resources available in the services. Women may not seek help, even though perinatal services have been developed and are available. Additionally, women may access helping resources but choose not to disclose their problems [5,8,18,19]. Research has shown that in the mental health context, persons seeking help might not exactly know what type of care is available and could help them, and they want to alleviate their psychological distress in whatever way they can [14]. Hadfield and Wittkowski [8] identified “an early stage of seeking help”, when women seek informal support from friends, family, and the Internet; in this stage, women recognize that something is wrong, but they do not yet seek help. Accessing treatment for mental health issues is delayed in many cultures [16,20], and many choose to solve their problems themselves [16,20,21,22]. Women may lack knowledge around mental health and service use, or they may encounter negative attitudes toward mental illness. Help-seeking in PPD is important during the perinatal period, because the primary caregiver and infant ideally form an attachment relationship during this time. If the primary caregiver takes account of their own and others’ mental states, an attachment relationship may evolve [19,23,24]. If women with PPD do not seek help, the infants will also suffer.

Existing reviews on help-seeking in perinatal mental health focus on overall help-seeking [25], on barriers to PPD help-seeking [5,8,18], depression [5,7,8,17], postnatal help-seeking [5,7,8,25], and peer support [17]. They primarily focus on help-seeking in services [4,5,7,8,18] or collect data in a specific country, such as the United Kingdom [4,18].

In existing reviews on help-seeking around perinatal mental health, mothers have revealed a range of emotions. Even negative experiences can be interpreted as normal symptoms related to pregnancy and motherhood [5,18,25], and mothers may choose to remain silent so as to not upset family members and to fulfill social expectations of good motherhood [4,5,8,17,18]. Family and friends are very important and influence women’s choice to seek help or not [25]. When seeking help, women feel their vulnerability will have negative consequences, such as being judged by others [25,26,27], creating guilt and shame [8,17,18] and a fear that the baby could be taken away [5]. The individual-level barriers to seeking help include poor awareness and knowledge about PPD [4,5,8,18,25] and a lack of open discussion between family members and professionals [5,17,18]. The mothers may be unable to articulate how they feel [4,5], be hesitant to admit their symptoms [5], or feel isolated [6,17].

As women do not easily reveal PPD and seek help [5], an integration of current knowledge on the topic is needed. We recognize a gap in the existing knowledge, on an international level, at the stage of contemplation in the perinatal period concerning psychological distress. Understanding women’s perspectives may guide the development of targeted support and interventions. Overall, help-seeking as a decision-making process versus as a multidimensional phenomenon draws our interest. What is needed is an integration of the body of knowledge related to women’s experiences in their everyday life at a stage when they contemplate whether to seek help.

### 1.1. Theoretical Perspective

A person’s life situation is central in making meaning of oneself. Relationships with others affect one’s general being in the world as well as one’s self-awareness [28]. This is an area that is very difficult to study and collect data on, since the complexity of the lifeworld and the lived reality is not necessarily recognized by the human herself. The existential context, the human history, and the society we live in shape our internal world of understanding, along with our feelings and thoughts [28]. Our study approach is informed by the philosopher Lauri Rauhala [29], who posited that experiences are labeled according to the meaning given to specific situations within the individual life situation. The unique life situation gives meaning to human existence [29]. However, the meaning may change as time passes [28]. We want to capture the perinatal period, which is considered a specific moment in time. Human experiences involve different levels of awareness or clarity, and the meanings individuals make are not always directly or clearly related to a specific phenomenon of interest [29]. Therefore, the idea of this study is to reach a description of the experiential level of women on psychological distress in perinatal help-seeking, including mental health issues such as depression, anxiety, mood disorders, and psychosis [2]. This experiential level may include emotions, sensations, experiences, description of everyday life events, and notions about life circumstances.

### 1.2. Aim and Research Question

The aim of this study is to integrate and synthesize knowledge of women’s experiences around contemplating help-seeking for PPD. The specific research question is: “What are women’s experiences of contemplating help-seeking for perinatal mental health distress?” The goal is to reach a new understanding and to develop practice by using a new innovative approach: to help women and their families, health care personnel (HCP) invite them to care services and discuss service options (psychiatric nurses in communities, public health nurses, midwives, etc.). The results can be used to develop a model of help-seeking in perinatal distress when mothers contemplate accessing mental health care. The focus is important from the perspective of families seeking help, as well as from the perspective of health and social care policymakers and professionals.

## 2. Materials and Methods

Meta-synthesis originating in meta-ethnography, as developed by Noblit and Hare [30], was chosen for this study; as a complex synthesizing process, it has the option of dealing with adverse data, is clearly structured, and can be used to gain understanding of meaning via interpretation [31]. Meta-synthesis also offers room for novel interpretation [32], openness, and creativity to gain new insights beyond the original research [31,33]. A meta-synthesis can facilitate gaining greater understanding in both depth and breadth than the findings of individual studies [34]. Noblit and Hare [30] suggested that rather than a predetermined framework, an inductive approach should be used to capture the uniqueness and meaning in a specific context.

We followed the eMERGe guidelines, which provide recommendations for conducting and reporting of meta-ethnography, developed by France et al. [31] (Table 1).

### 2.1. Search Strategy and Criteria

**Figure 1:**
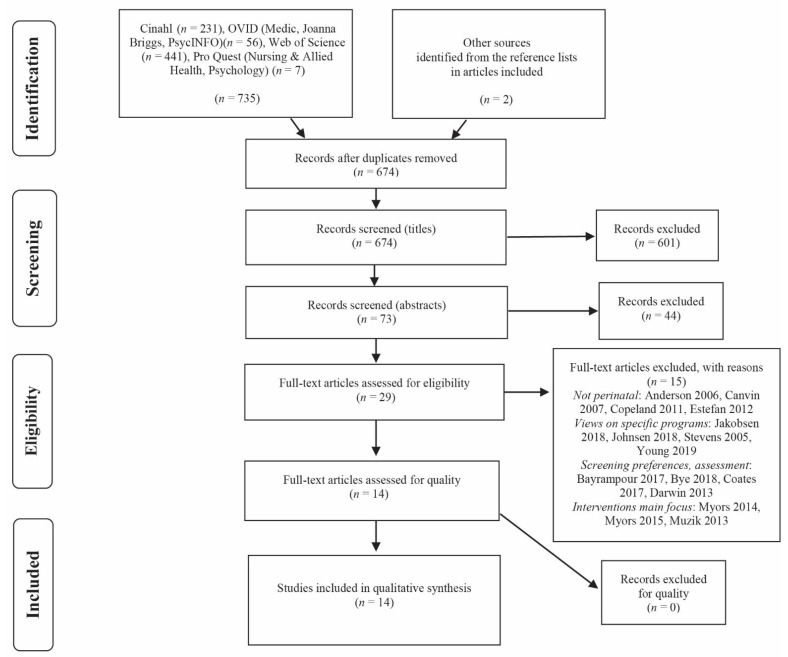
The PRISMA flow chart of the current study.

We conducted an extensive search in December 2019 with the aid of a librarian, who assisted us with database selection and choice of terms for the search. We used the PRISMA flowchart [35] to describe the process of selecting relevant studies for the meta-ethnography (Figure 1).

The search was completed through separate steps, which were combined at a later stage. The terms were searched in titles and additionally as MeSH terms were:

- mental disorders* OR help seeking behavior* AND mothers* OR maternal
- help-seeking behavior OR health care seeking behavior OR health behavior OR acceptability of health care OR patient acceptance of healthcare OR services utilization OR health care utilization OR health seeking OR health care seeking behavior OR perceived barriers OR barriers to OR facilitators to OR perceived access OR perceived barrier OR client participation OR client engagement OR client involvement

We intended to include all types of mental illness and disorders:

- mental disorders OR mental health disorder OR mental health or anxiety OR anxiety disorders OR depression OR depressive symptoms OR mental illness OR psychiatric and anxiety OR psychotic disorders

As the focus was on the perinatal period, we also included the terms:

- pregnancy OR infant OR baby OR child OR perinatal time. Finally, we wanted to focus on qualitative studies, so we combined the previous searches with AND qualitative studies OR qualitative research OR phenomenology. We did not include alternate spellings such as ‘utilisation’ in addition to ‘utilization’.

The inclusion criteria: perinatal period from women’s perspective, primiparous and multiparous, peer-reviewed publications, published in English, age of women over 18 years, and research from Western cultures.

The exclusion criteria: women and girls under 18 years, quantitative studies, reviews, RCTs, protocols, child-related problems during the perinatal period, focus on fathers, immigrants seeking help, focus on physical health, staff perspective on women’s help-seeking.

We did not set a time limit in the original search: our idea had been to include wide ranges of mental health questions around PPD, including dual disorders and substance-related questions, yet we removed early entries from our search terms, and as such articles were scarce. By the stage of reading the full texts, we identified studies outside the perinatal period and excluded studies that described single interventions, feasibility studies, screening tools, or perinatal services (programs). As cultural context impacts help-seeking and experiential stigma of mental illness [1,4,5,18,25], we focused solely on Western societies, and we acknowledged the differences, such as those in the healthcare systems in specific countries. EndNote online software (Clarivate Analytics, Philadelphia, PA, USA) was used to facilitate the process of choosing relevant references. The search and inclusion strategy are presented in Figure 1.

The first author screened the titles first, and then the abstracts. All authors were involved in developing the data screening strategy and in screening the full texts. Disagreements among the reviewers about inclusions and exclusions were resolved by discussion and finding consensus. The inclusion and exclusion criteria required refinement throughout the steps (see Figure 1).

### 2.2. Qualitative Appraisal of the Studies

We used the Critical Appraisal Skills Programme Qualitative checklist (CASP) [36] to ensure quality assessment of the studies. Two researchers read the articles independently, then evaluated and scored the studies (yes = 2, to some degree = 1, and no = 0). The CASP evaluations were discussed together in the study group, and the average sum was used as the basis of scoring. The maximum CASP score was 20, and the included articles were rated in the range of 13–20: no articles were excluded. Most scores were lowered by the CASP question “Has the relationship between researcher and participants been adequately considered?”: only 4/14 articles received full grading. The CASP scores can be seen in Table 2.

### 2.3. The Interpretation and Synthesis Process

**Figure 2:**
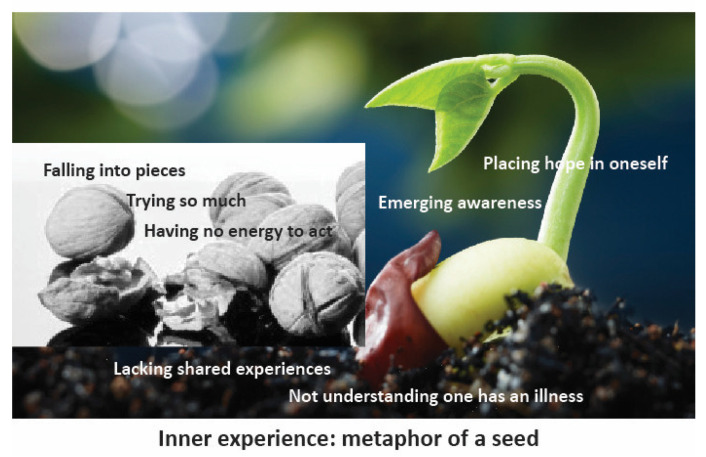
The inner experiences of women with perinatal psychological distress contemplating help-seeking.

The eMERGe steps for the data comparison [31] began with determining how the studies are related, which required several readings. We made an initial assumption about the relationship between the included studies being analogous [30]. We performed data extraction using first- and second-order concepts, independently in pairs by listing the findings using line-by-line coding [31]. The meaning units were discussed in the research group and thematized (see Supplementary File, Table S1 for all themes extracted). The first author maintained an overall view by participating in both pairs. Translating the studies into one another was meant to compare the findings from one study with those from another. This step was also done in pairs, and discussions led to consensus. The themes were clustered and reflected on (see Supplementary File, Figure S1 for preliminary clusters). The relationships among the clusters were reflected on and discussed in the study group. We went back to the original studies for confirmation and understanding. QSR NVivo software (QSR International, London, UK) was used to facilitate the process. Synthesizing translations meant that we analyzed the translations, thus going beyond the findings of the individual studies to a second level of synthesis [30,31,33]. All authors were involved in the process of searching for refutational findings. A lines-of-argument synthesis based on the metaphorical themes (Figure 2) was created through an in-depth, back-and-forth, iterative analysis process between the translations and the articles.

## 3. Results

All study characteristics of the 14 studies included in the meta-ethnography are presented in Table 2, with information about the author, year, country, aim/objective, participants, type of psychological distress and inclusion criteria, setting, data collection method, results, and CASP evaluation. Over a third (36%) of the studies were from either the United States or Canada, two (14%) were from Australia, and one (7%) was from New Zealand and the United Kingdom. The publications dated from 2002–2018.

The total number of participants in the included studies was 345 mothers (range 18–52 years, 25 years on average). The age of the women was over 18 in all studies, although the age was not known in two studies. Most studies described women suffering from depression (71%); one study (7%) included women with bipolar disorder, one (7%) with postpartum mood disorders, and two (14%) women with more general mental health problems. A majority of the studies (57%) focused on postnatal depression or on prenatal depression (14%). Usually, screening was used to determine participants’ inclusion in the studies, with the most common assessment being the Edinburgh Postnatal Depression Scale (EPDS). The settings and contexts for data collection varied: mental health settings were used in three studies (21%), and obstetrics settings, midwifery care, public health settings, well-baby clinics, or parent resource centers in the majority of studies (50%). Two studies (14%) collected data through a combination of tertiary care and mental health services. Two studies (14%) collected data in internet discussion groups for mental health concerns.

The data collection tools used were interviews (43%), focus groups (29%), a combination of interviews and focus groups (14%), and open-ended questions in an online forum (14%). The analysis methods varied as well, including content analysis (29%), grounded theory (29%), thematic analysis (21%), and phenomenology (14%). One study (7%) was a mixed-methods study, with the qualitative analysis facilitated by grounded theory with a phenomenological emphasis.

Women’s experiences of contemplating help-seeking for PPD involve a time period of existential turmoil without a solution. Unresolved PPD consists of aspects that may exist separately or simultaneously, and no linear process could be detected. These aspects were: ‘Falling into pieces’, ‘Trying so hard’, ‘Having no energy to act’, ‘Lacking shared experiences’, ‘Not understanding one has an illness’, ‘Emerging awareness’, and ‘Placing hope in oneself’ (Figure 2). We did not observe a straightforward and linear process, as previous research had suggested, but rather a multidimensional and ambiguous life-situation wherein women try to solve their situation.

### 3.1. Falling into Pieces

Contemplating help-seeking in PPD was a time of extreme experiences, with one aspect being ‘Falling into pieces’. Being very tired all the time might lead to others claiming it is a normal state with a new baby, even though the women were at an extreme [37]. They felt overwhelmed by emotions, lack of functioning [37], and mood changes [38]. They might have experienced joy for a few days, but changed into losing control of their lives, a comparison to life prior to the baby was born, and struggling with the “loss of life as it used to be” [39]. Women felt they had entered a downward spiral, where experiences added on to one another [39,40,41]. Women went through a range of emotions [38]: feeling helpless [37,42], feeling hopeless [42], being more negative [39,41], dwelling in anxiety [37], being irritated, frustrated, and angry [38], or presuming they must be lazy [42]. Losing control appeared with a fear of ‘going crazy’ and ‘panicking’ [42].

“Problems quickly generalised to all aspects of infant care: ‘once you failed at one thing, for example you thought you’d failed at your breastfeeding, you then decided you were going to fail at everything. So it kind of just circles off’.”

[39]

Experiencing sadness felt contradictory, such as when the home or baby were beautiful and life should have been enjoyable [39] or when one should be grateful for pregnancy [27]. Women with previous mental illness were able to analyze their condition and make decisions about when help-seeking was ultimately required [41,43].

The emotions related to their infants were manifold, with anxiety about being alone with the baby [40], feelings of not loving the baby [37], and even thoughts of hurting the baby [42]. The irrational thoughts they experienced also impacted other children in the family:

“I was afraid to show my new baby any affection in front of my toddler for fear that she would think I didn’t love her anymore.”

[42]

Women felt they were unable to care for their baby [37,42]. Some women expressed that they spent their days doing nothing but crying [42,44], and they experienced a sense of failure at parenting [39]. If they noticed their actions in relation to their infants were out of control and contrasted with their ideals [42], the anxiety with the baby and the experience of falling into pieces might lead to help-seeking. However, the experience of shame and guilt might inhibit them seeking help [40,41,42,45] until their condition was no longer manageable [41]. When they could not take it anymore, they sought help after months of suffering [42].

Women were “their own enemy and barrier for support” [40]. The worst situations were dramatic events, where even police and emergency health care were involved, and the women were forced to go to the hospital [46]. Ultimate crisis points included suicide risks [27,37,38,40,42,43,44].

### 3.2. Trying so Hard

The theme ‘Trying so hard’ describes women trying to adjust to external norms while also trying to solve the situation, surrounded by the opinions of their loved ones, and pondering whether a change was needed. The confusion was ongoing and related to social expectations of what was required at the time of being a new mother: it seemed that external expectations/norms suggested that denial of one’s own feelings was favorable. Women may struggle to admit there are problems [47], and they can blame themselves for being “dramatic” [45] or “weak and stupid” [42]. The women had high expectations of themselves: they were proud, and they felt like they were failing in parenting. They did not want “to be seen as a failure”, and they blamed themselves when they were not able to live up to their own expectations or those of others [39,45]. In the eyes of others, the women should keep up appearances [39]. The need to be viewed as the “perfect mother” motivated many women to mask or deny their condition [40]. Women said their family members’ obliviousness of the fact that things were not right made the women lie to family members [39]. According to cultural and social norms, motherhood should be a joyful and happy time in life [42,48], yet the women did not seem to be able to perceive their life situation in the same way as the cultural expectations around motherhood suggested [48].

“I’d be told it was normal, that it was fine, that everybody felt that way, that it shouldn’t matter because I should just be happy that I have a healthy baby.”

[48]

Women reported that HCPs or peer groups had “normalized” or minimized their condition [27,37,38,40,44,48]. Their conditions were dismissed by HCPs as normal for pregnancy or due to “pregnancy hormones” [27], with a view of “pregnancy as a time of mental health challenges” [38] or the situation was labeled as “having postpartum” [41], “adjustment to motherhood”, or the “baby blues” [44]. Their condition and symptoms were not severe enough and might have been explained by other circumstances [37]. Thus, women believed their explanations were not taken seriously, and they themselves also tried to normalize their experiences [44]. “Women should not need emotional support during pregnancy or the postpartum period” [45]: expressing their concerns to loved ones may result in invalidation. Women were left feeling shaken and forced to manage their condition on their own [41]. For others, however, “normalizing” meant making the difficult questions easier to discuss [41]. Some women just needed to talk about the different feelings and to accept them [49]. Talking about something that was considered normal for the life situation was considered safer than the risk of mental illness, which had a scary feeling [41]. Women struggled with the distinction between “normal” feelings of distress and exhaustion versus the distinction of an overwhelming condition that might justify seeking help [37].

“I even went in at 3 months and I talked to a health nurse, and I just lied through my teeth because I thought, “What are they going to do if they find out I can’t be a good mom?”

[40]

External expectations have an impact on creating or enforcing direction. Social norms, with beliefs and attitudes on both intensive mothering and mental illness, created situations where seeking help was not an option [39,42]. In contrast, some women said that normalizing lowered the threshold for seeking and receiving help [41]. Given the impact of cultural and social factors, the women may not have been able to perceive personal needs or make choices based on their own perceived wishes [48]. The negative experience of other peoples’ viewpoints was called stigma, and women feared being stigmatized by others [40,41,45,47,48,49]. Stigma included the labels of “being a bad mother” [39], “being disorganized and unmotivated” [37], “being lazy” [42], and showing that “women could not cope” [40,41,43].

PPD led to labels related to diagnoses [41,45]: women feared that a statement in the records would follow with a label of a psychiatric patient [47]. Women also expressed a fear of medications [43]. In general, PPD could be denied with an expectation that “it can’t happen to me” [39], or PPD made the mother feel like a “second class citizen” [27]. They did not want others to know that their family was imperfect, and thus self-blame ensued [45]. The idea of experiencing mental illness caused feelings of guilt and shame [39,40,41,42,43,47,49].

Women said they tried or even “tried hard” to obtain help from HCPs [27]. Authors explained that some mothers had the ability to seek help [42,46,48]. Women had to strive in midst of their sense of self-agency and cultural pressure.

### 3.3. Having no Energy to Act

Women reported feeling overwhelmed by their emotions: “everything just got too much for me, and I couldn’t go anywhere to get any help”. Consequently, motivation, along with the strength to make decisions, were lacking [39]. The women said that one reason for not seeking help was an experience of exhaustion: they had no energy to seek help [37,38,40,42,48]. Women spent the whole day at home dressed in nightclothes [42], experiencing fatigue, anxiety, and stress around leaving the house and keeping appointment times [48].

“When I was experiencing mental health issues, it was harder for me to get out, sort of on a schedule and be punctual.”

[48]

Leaving the home was difficult with a small child [47], because it required so much effort [40]. Difficulties were faced, as services are run on a fixed schedule, which requires mothers to travel with their newborn to these locations outside their home [48]. Women reserved appointment times and yet canceled appointments [42]. As women did not have sufficient information about available services, seeking care also required having the energy to identify what resources existed [48]. With their exhaustion and fatigue, the women simply did not have the energy to act.

### 3.4. Lacking Shared Experiences

Many women reported loneliness [40,42,43,47]. Some women saw themselves as weak if they were not managing alone at home [45]. They felt isolated [38,40,42,47], felt alone [40], felt worthless [40], and withdrew from contacts [40]. The isolation was related to a view of oneself being a private person, who kept a lot inside and only opened up to a handful of people [42]. Others imposed isolation from friends and other mothers on themselves [37]. A lack of support prevented women from seeking help [45], while others expressed that they felt unworthy of support [40] and lacked trust [49].

“I still felt so alone as nobody in my close knit group of friends and family had ever had gone thought this and could not truly relate.”

[42]

Thus, many women described a lack of shared experiences [42]. They felt they could not speak to anyone [37], and many women did not have supportive relationships to rely on [38]: they described limited or absent friend and family networks, which was worsened by having unsupportive or absent close relationships lacking sufficient communication or responsiveness [38,40]. Childless friends did not understand, and friends with children were too busy [40].

“I ask my husband for help, and he tries for maybe two days, then it’s right back to being unhelpful and uncaring.”

[42]

Others wanted more help and support from their partners [27,37,40,45,47]. Women appreciated caring partners [38,42,43,48] and instrumental support from friends [40]. They wanted proactivity, such as friends or partners intervening when they saw that help was needed rather than waiting for the mother to ask for help [40]. A lack of support from their spouse or partner contributed to making it even more difficult to seek help [45], as their partner often discouraged them from seeking help because they “just need to calm down and stop crying” [45]. The changed situation—with a newborn baby and mental illness—put a strain on their relationship, which was at times difficult to emotionally and physically sustain [39]. Some women with unsupportive partners ended the relationship within weeks or months of the baby’s birth [43]. Conflict with a partner may have existed prior to childbirth, or it may have worsened due to the mother’s mental health struggles [43]. Some women blamed their partner for hindering help-seeking [38,40,42].

As women lacked shared experiences, their actions as they tried to seek help may have seemed chaotic: women struggled to decide whether to seek help [47], and those who sought help expressed that it was difficult to find [39]. Women did not know where to seek help [43] or from whom to seek it [41]. A gap in knowledge seemed to exist, since women were unaware of what services were available and for what [45], and they did not understand how hospitals could help or what they could expect from medications offered to them [43,45].

“I don’t think I sought outside help because I didn’t know.”

[43]

Some women sought for information and emotional support help online, via Internet searches and new-mothers websites [40]. One source of knowledge about treatment options was media [39,46].

“The participant developed negative beliefs about the side effects of medications. Whether or not the information gained by participants through experience with family members or the media is accurate, it influenced their beliefs about postpartum depressive symptoms and possible treatments.”

[46]

A lack of shared experiences resulted in less reflection on one’s own life situation and condition. Disclosing their thoughts or reaching out was an issue for many mothers [47], since they believed they could not talk, felt uncomfortable talking [37,42,48], or felt that discussing mental illness was troublesome [44].

“There’s the fear of really telling the truth and I felt safer telling the midwife than I did telling my doctor the depth of how I was feeling because I was really scared and I was having pretty awful thoughts and even though I was seeing them both at the same time and they were both referring me, I didn’t really open up to my doctor and tell her the truth if you know what I mean?”

[48]

Even though mothers shared that talking with HCPs and others about their distress and experiences was perceived highly significant in the recovery process [37], they did not disclose their thoughts to HCPs if they risked being criticized, so they waited for a suitable time to talk [27]. Women wanted compassion and care, but they were not always successful. Women said their experience of depression was a low priority for health professionals [27]. If a woman had hope for communication with an HCP and noticed that the HCP did not understand, she may have experienced the communication as insensitive and may not have felt cared for [47]. Women seemed to avoid conflict and chose to believe that time would heal [44,47]. Lacking shared experiences made help-seeking intentions challenging, because of how they interpreted care encounters with HCPs.

### 3.5. Not Understanding One Has an Illness

At a certain stage, women noticed that something in their everyday life experiences had altered, yet they did not have a resolution at hand. The situation, emotions, or behavior might have become difficult to explain, and even if describing it was possible, they could not name the experience [37,40]. Women noticed they were not themselves, thinking clearly and logically was challenging, and a lack of motivation emerged [39]. Without knowledge and understanding, the identification of an illness was not possible [37,44,48], and they were confused as to whether depression should be regarded as normal or not normal [37]. Losing interest in everyday life activities (such as showering) felt confusing [42].

“I just didn’t know what I wanted at the time and I didn’t know what I wanted to get out of it. I didn’t know what was going on.”

[47]

The life situation might have been experienced as unclear: some reasoned that it was due to being tired and that the lack of clarity might be related to major sleep deprivation [37].

Some women discussed with their friends, and even though close relations suggested whether the strange feelings could be depression, the women might be reluctant to take the advice [37]; additionally, the expressed worry and concern was not always taken as a sign of illness or the need of HCP involvement [44]. Others reacted when their partner, friend, or close relative said “You’re not yourself” [40].

Mothers did not recognize that they were depressed until they could not take it anymore [42] or their mental illness was later identified by a health professional [37]. PPD can be an unclear condition from the perspective of the new mother.

### 3.6. Emerging Awareness

The women described awareness of their own life situation as though it was emerging via several steps, observations, and perceptions, as well as living through the situation. At the start, women may not have known that the changed life situation was such that they could seek help to solve the situation [48]. Emerging awareness was described in several studies [27,40,41,42,44,45,46,47,48]. It included recognition of behavioral changes, which indicated mental distress [46], and awareness of their own condition [44].

The recognition of the changes may have arisen via a close personal relationship [46] —e.g., a partner or relative labeling their experience as mental distress. The woman could then note that the suggestion of a relative prompted her to consider whether she was suffering from postpartum depression [46]. Another point of recognition was when women recognized and became aware of “irrational thoughts and crippling guilt” [42]. Women may not be aware of the varying degrees or different symptoms, which do not fit everyone, and therefore delay their help-seeking [48].

“It’s as though you have to figure it out for yourself first before you actually get the resources you need.”

[48]

Other family members were unaware of what was happening [43] and of the magnitude of women’s problems [40] until hospitalization occurred. If family members were not aware of mental illness or did not note that some action in relation to help-seeking might be needed, the mother was not supported [40,42,47]. Without knowledge of PPD, family members may minimize the situation by attributing the condition to other factors, such as lack of sleep [40,42], which may deeply contradict the women’s own experiences. As women may want to hide or not disclose their situation, family members do not always have the opportunity to be supportive [43]. Some family members helped mothers extensively, such as a mother’s mother moving in for a month or the partner waking up at night to feed the baby [42].

Speaking and reflection may be a way to raise awareness [42,47]. Reading and obtaining more information [47] can be experienced as empowering if the mother learns more about illness, the importance of treatment, and the availability of helpful resources [45]. However, other women did not seek information [46].

“The knowledge of mental health. I think it is probably the biggest thing, knowing, recognizing the signs and symptoms. Just knowing that I needed to seek help before it got worse.”

[45]

A partner’s engagement in discussion and learning about perinatal mental health was highly appreciated and might have impacted women [38]. Recognition of stigma around mental health issues may have opened the women’s eyes to seeing their lack of understanding and awareness of depression during their pregnancy [27]. In the situation of PPD, the continued discomfort creates a need for change and a search for solutions, which may or may not include informal or formal help-seeking. Women try to solve the inner conflict by various means.

### 3.7. Placing Hope in Oneself

The mothers made choices themselves and relied on themselves [38,40,42,44,47,48]. Relying on the self was seen as important in identifying how to address one’s mental health needs [38], as women needed to place hope in themselves.

When some women relied on themselves, they did not rely on others to take care of their babies:

“I had to do it all on my own, because nobody could do it well enough. I had a lot of anxiety. I didn’t want anybody holding him, I didn’t want anybody touching him. I didn’t want anybody in my house, because if I turned around and left him alone for a second, somebody would hurt my baby.”

[40]

The mothers tried to handle their situation through positive self-talk, adhering to daily routines [40,48] or journaling [38,48]. Guy et al. [46] shared actions related to self-help: leaving the house; taking time for oneself; letting emotions out through activities like crying, using substances, and eating comfort foods. Women used actions related to making changes in family functioning by asking family members for help or implementing a schedule for the children. They also pursued change by seeking employment or attending religious events. Some of the listed self-help options, such as using substances, were actually self-destructive in the long run [46].

The mothers sometimes decided to wait for the situation to improve by itself [44]. In group discussions, women shared that they “just did not like to be supported” [43]. Relying on partners, friends, and service providers was the next best option [40].

### 3.8. The Metaphor of a Seed

Contemplating help-seeking for PPD may be understood as a life situation in turmoil, as mothers make meaning in a specific life situation or context where several levels of experiences coexist, and the women contemplate whether to seek help. We have interpreted this as the metaphor of a seed describing symbolically the perinatal mental health time period with psychological distress as a multidimensional concurrent life situation: ‘Falling into pieces’, ‘Trying so hard, ‘Having no energy to act’, ‘Lacking shared experiences’, and ‘Not understanding one has an illness’ may result in ‘Emerging awareness’ and ‘Placing hope in oneself’ (Figure 2).

The metaphor of a seed represents an interpretation of the phase prior to seeking help. As seeds need to have suitable conditions for growth, help-seeking for PPD involves the inner unresolved individual experiences. Individual experiences manifest as a symbolic seed, at risk of being cracked into pieces by the surrounding circumstances and inner experiences. In describing the situation of women experiencing PPD, the metaphor of a symbolic seed also describes women’s own resources and possibilities. The solution lies within the women themselves, as emerging awareness and hope may co-exist or evolve. Each of the identified themes involves ambiguity and emotional turmoil—an unresolved life situation. Women try to solve the inner conflict through different means. They experience experiential turmoil as a metaphor of falling into pieces, and they may not have the words to express their needs. Since women may experience inner isolation and they do not have the strength or knowledge to seek help, they may not be able to seek help. Their solution may be to continue to solve the situation themselves, but they may not have the opportunities or strength to resolve the uncomfortable situation. This is why family and other loved ones play a crucial role along with HCPs.

## 4. Discussion

We have identified a gap in the existing help-seeking literature of the step prior to seeking help. We call this contemplating help-seeking, and we approached the phenomenon with a perspective inspired by the philosopher Lauri Rauhala [29] and the nursing researcher Karin Dahlberg [28]. We chose to focus on women’s lived experiences, the experiential level—including emotions, sensations, and perceptions—which may involve experiences of different levels of awareness and clarity, and the meanings can be ambiguous. In a meta-ethnography, new interpretations can be revealed in the qualitative evidence synthesis through the identification of new approaches and viewpoints [30,31].

Studying help-seeking for PPD is important, because previous research has shown that help-seeking is faced with delays [20,21]. Even though women participate in and access health or social services and programs, they may not disclose their problems [5,8,18,19]. Contemplating help-seeking in the perinatal period is important to a health policy perspective, because PPD may impact parenthood, be reflected in a person’s everyday life, and have an impact on an attachment relationship being formed between the parent and the infant [19,23,24]. In the phase of contemplating help-seeking for PPD, a focus on only the mother needs to be replaced with a focus on mother–baby relationships and family aspects.

In existing help-seeking theories, a cognitive level is the primary focus, and help-seeking research is based on perceived needs, viewing persons as rational decision-makers and action-takers when they decide to seek help [1,13,14,15]. We did not find such a phenomenon; instead, we identified a layered and multidimensional life situation. Pregnancy, birth, and becoming a mother may collectively represent a critical period of physical and emotional upheaval in a woman’s life [41]. An intentional human being performs goal-directed tasks, but with mental health-related issues, such as PPD, the intentions may be out of the person’s reach. According to Viveiros and Darling [48], the ability to perceive the need for care is determined by health literacy, knowledge about health, and beliefs related to health and sickness. Previous reviews on perinatal mental health showed poor awareness of and insufficient knowledge on PPD [4,5,8,18,25]. Women may be unable to recognize or express their emotions [4,5]. Through our theoretical perspective, we see that humans create meaning in the context of their individual life situation, where the present and the past impact current meaning-making, and we can assume that humans grow in relation to each other [28,29]. Our results showed that women wanted to be sensitive toward their family members: this might lead to a lack of open discussion in the family or women not wanting to upset family members [4,5,8,17,18]. Given that women seem to want to protect their loved ones and are sensitive to critique, societal stigma around mental illness may be especially harmful. Therefore, it is essential to develop information and provide education for society around mental health-related questions and make such information available for women by pregnancy at the latest.

Women as individuals experiencing situations of seeking help around mental health issues try to solve burdens their own way. Isolation was identified in our meta-ethnography, as it was found in previous research [6,17]. We identified a lack of recognition and support, and lacking shared experiences, why it is especially important for family members to acknowledge the mothers need for support, also in a proactive way. Many mothers are unwilling to disclose their situation, due to emotions of shame and guilt [8,17,18] or being judged by others [25,26,27], as the expectations of them seemed to involve being “supermoms”. Our findings add to this by synthesizing the complexity of PPD and by identifying a time period of existential turmoil without a solution. The non-linear themes involving unresolved questions during PPD consist of aspects that may exist separately or simultaneously: ‘Falling into pieces’, ‘Trying so hard, ‘Having no energy to act’, ‘Lacking shared experiences’, ‘Not understanding one has an illness’, ‘Emerging awareness’, and ‘Placing hope in oneself’ (Figure 2). In describing the situation of women during the perinatal period, we established the metaphor of a seed. Individual experiences manifest as the seed, at risk of being cracked into pieces by the surrounding circumstances and inner experiences. In describing the situation of women experiencing PPD, the metaphor of a seed also describes women’s own resources and possibilities. Each theme involves ambiguity and emotional turmoil—an unresolved life situation. Help-seeking actions are possible in all these phases, possibly resulting in change. Women try to solve the inner conflict through different means and endure a lot before a solution starts to emerge. During this time period, they are especially vulnerable and susceptible to the viewpoints and advice of their loved ones.

### 4.1. Implications for Practice

If the HCPs recognize the phase of contemplating help-seeking, HCPs can address women’s concerns seriously prior to clients accessing services. Women with PPD during the perinatal period may not recognize an illness, perceive a need, name a disorder, or search for suitable care providers. Women may not view their life situation as a barrier to care from HCPs but rather try to manage their life situation in the best possible way. As the perinatal period is a unique time during women’s and infants’ lives, our results question how women are approached by health and social care services and programs. It has been indicated that HCPs should develop their skills at an attitudinal level, as well as from the perspective of how to approach women with mental illness during the perinatal period. Mothers with PPD lacking shared experiences need an especially sensitive and appreciative manner of encouraging and enhancing reflection, possibly supporting the close relatives. Understanding mothers’ contemplating help-seeking for PPD in more detail would enable the engagement of whole families with services. Understanding more might also counteract women dropping out of treatment before even starting to use the services.

As women’s inner possibilities and awareness have a crucial impact on their decision to seek help, finding solutions that enhance their inner capacities and experiences of hope in the current life situation is important. Contemplating help-seeking for PPD may be connected with empowerment and growth in the role of mothering, as maternal well-being is a prerequisite for the health of the children. HCPs might use reflexive tools to assist in co-creating awareness and to implement a positive approach. We suggest that existing strategies and recommendations on perinatal mental health issues would benefit from the micro perspective, as women can only be helped by services if they themselves are willing to participate and share their lifeworld with those helping them in the services. In our study we focused on mothers, yet the study of contemplating help-seeking from the fathers’ perspective would be important as well.

### 4.2. Strengths and Limitations

The chosen search strategy was useful, as we reached a sufficient amount of data and excerpts to support a robust analysis [31]. The studies included were versatile in many aspects, which is a strength of meta-ethnography. We used the CASP qualitative appraisal tool [36] for quality assessment of the studies.

As our focus was on PPD, most of the studies included in the meta-ethnography focused on depression [27,37,38,39,40,42,43,44,45,46,47,49]. Only a few studies described other types of psychological distress, such as post-partum mood disorders [41] or bipolar disorders [45]. Viveiros and Darling [48] discussed perinatal mental health, which included depression, anxiety, and other mental health concerns; Raymond et al. [38] discussed “a range of emotional and mood challenges”. We did not identify any studies on women with psychosis seeking help during the perinatal period. The area of PPD is so wide that we can see that our data were restricted; on the other hand, the current focus on PPD enhanced our interpretations, and our sample was versatile in many ways (Table 2), which is preferred for meta-ethnographies [31].

Research in studies related to mental illness has its restrictions, since researchers often choose a problem and illness paradigm, such as in the purpose of delineating the study purpose. In our search terms, we used diagnostic labels. This is a limitation, since the search terms and aim of the study did not focus on aspects such as positive mental health [50]. In our data, ‘Placing hope in oneself’ included both the active stance of oneself as well as women trying to solve issues through self-help means and by identifying positive aspects as self-care. Even in the most difficult life situation, with mental illness and strain, the positive approach of mental health can also co-exist [29,50]. The philosopher Lauri Rauhala explained that experiences like unpleasant life events are labeled, and the unique life situation gives meaning to human existence [29]. The positive aspects may be relevant in the meaning-making of the everyday life situation. From a lifeworld perspective, the lived reality is not necessarily recognized by the human herself, which is why researchers must be aware of how their approach impacts the study results. Using the lifeworld perspective in this meta-ethnography proved to be productive. Using a metaphor with a picture to visualize the interpretation of the inner experiences can open understanding, and may however, also be considered a novel way of scientific description of phenomena [28,29]. The interpretation is connected with the Results section as a whole and will need to be read in connection with the multidimensional themes.

## 5. Conclusions

The meta-ethnography in this study provides a thorough picture of contemplating help-seeking as the phase prior to seeking help in perinatal psychological distress. We did not observe a straightforward and linear process (as previous research suggested) but rather a multidimensional and ambiguous life situation, where women try to solve their situation and lack skills that promote help-seeking. Women with perinatal psychological distress try to solve their situation first by themselves; their life situation is such that help-seeking may not be an option, even though helping resources are available. A clinical implication to improve practice, policy, and service user outcomes in health and other fields is that service providers should work with outreach and develop tools to connect with these mothers. HCP’s need an especially sensitive and appreciative manner of encouraging and enhancing reflection, possibly supporting the close relatives, since mothers contemplating help-seeking for PPD may lack shared experiences or have the skills for reflection. Another suggestion is to improve training in mental health literacy prior to or during pregnancy.

## Figures and Tables

**Table 1:** The implemented eMERGe Reporting Guidance in this study [31].

Criteria Headings		Reporting Criteria	Page
Phase 1 Selecting meta-ethnography and starting	1. Rationale and context for the meta-ethnography	Describe the gap in research or knowledge to be filled by the meta-ethnography, and the wider context of the meta-ethnography	1–3
	2. Aim(s) of the meta-ethnography	Describe the meta-ethnography aim(s)	3–4
	3. Focus of the meta-ethnography	Describe the meta-ethnography review question(s) (or objectives)	3
	4. Rationale for using meta-ethnography	Explain why meta-ethnography was considered the most appropriate qualitative synthesis methodology	3-4
Phase 2 Deciding what is relevant	5. Search strategy	Describe the rationale for the literature search strategy	5–7
	6. Search processes	Describe how the literature searching was carried out and by whom	6-7
	7. Selecting primary studies	Describe the process of study screening and selection, and who was involved	6–7
	8. Outcome of study selection	Describe the results of study searches and screening	6
Phase 3 Reading included studies	9. Reading and data extraction approach	Describe the reading and data extraction method and processes	15
	10. Presenting characteristics of included studies	Describe characteristics of the included studies	8–14, 15-16
Phase 4 Determining how studies are related	11. Process for determining how studies are related	Describe the methods and processes for determining how the included studies are related: Which aspects of studies were compared AND How the studies were compared	15
	12. Outcome of relating studies	Describe how studies relate to each other	15
Phase 5 Translating studies into one another	13. Process of translating studies	Describe the methods of translation: Describe steps taken to preserve the context and meaning of the relationships between concepts within and across studies Describe how the reciprocal and refutational translations were conducted Describe how potential alternative interpretations or explanations were considered in the translations	15
	14. Outcome of translation	Describe the interpretive findings of the translation	15–22
Phase 6 Synthesizing translations	15. Synthesis process	Describe the methods used to develop overarching concepts (“synthesized translations”) Describe how potential alternative interpretations or explanations were considered in the synthesis	15–20
	16. Outcome of synthesis process	Describe the new theory, conceptual framework, model, configuration, or interpretation of data developed from the synthesis	21–22
Phase 7 Expressing the synthesis	17. Summary of findings	Summarize the main interpretive findings of the translation and synthesis and compare them to existing literature	22–23
	18. Strengths, and limitations	Reflect on and describe the strengths and limitations of the synthesis: Methodological aspects: for example, describe how the synthesis findings were influenced by the nature of the included studies and how the meta-ethnography was conducted.	23–24
	19. Recommendations and conclusions	Describe the implications of the synthesis	22–24

**Table 2:** Characteristics and CASP scores of included studies.

Author(s), Year, Country	Aim/Objective	Participants	Psychological Distress and Inclusion	Setting	Data Collection, Method	Results	CASP Qualitative Evaluation
Bell, Feeley, Hayon, Zelkowitz, Tait et al. (2016) Canada	To explore perceived barriers and facilitators to the use of mental health services experienced by women and their partners.	30 women, 32.5 years (average)	Postnatal depression, inclusion EPDS * 12	Two hospitals providing tertiary care and mental health services	Interviews with couples Content analysis	Five principal barriers and facilitators: accessibility and proximity, appropriateness and fit, stigma, encouraged to seek help, and personal characteristics.	20
Bilszta, Ericksen, Buist and Milgrom (2010) Australia	To explore barriers to care by asking women who are experiencing postnatal depression (PND) and who have accessed treatment and support services; how they recognized and acknowledged their depression; how being depressed affected their ability to actively seek help; what sort of help they wanted and why and how the attitudes of health professionals, friends and family, and the general community influenced the type of treatment sought.	37 women, 34 years (mean)	Postnatal depression, EPDS * 14 median, most participated in structured treatment program	Hospital outpatient postnatal depression programs, community based mutual support programs	Focus groups Interpretative phenomenology (Smith)	Findings suggest the lived experience of PND and associated attitudes and beliefs result in significant barriers to accessing help. Eight theme clusters were identified: expectations of motherhood; not coping and fear of failure; stigma and denial; poor mental health awareness and access; interpersonal support; baby management; help-seeking and treatment experiences and relationship with health professionals.	13
Byatt, Cox, Moore, Simas, Kini et al. (2018) United States	To elucidate in a sample: (1) the challenges associated with under-recognition of bipolar disorder in obstetric settings; (2) what barriers they face when trying to access psychiatric care; and (3) their perspectives regarding how obstetric practices can facilitate the identification of bipolar disorder in this population and connect women with mental health care.	25 women, age 18–55 years	Bipolar disorder, inclusion EDPS * 10 and DSM-IV criteria for bipolar disorder I, II	Five obstetrics practices, tertiary care center	Mixed. Qualitative study interviews Qualitative study modified grounded theory with phenomenological emphasis	Participants want their obstetric practices to proactively screen for, discuss, and help them obtain mental health treatment. Most were unaware of their diagnosis. Self-blame, stigma, fear, and lack of support prevent women from seeking help.	15
Foulkes (2011) Canada	To explore the barriers and enablers identified by women experiencing a postpartum mood disorder (PPMD) that preclude and facilitate their help-seeking behaviors.	10 women, age 32.5 (mean)	PPMD, inclusion with no preexisting psychiatric illness and a diagnosis of postpartum mood disorder	Well-baby clinics and a parent resource center	Interviews Grounded theory (Strauss and Corbin)	The core category of ‘‘having postpartum’’ captured the essence of women’s experiences in seeking help for a PPMD. Women identified four main stressors that contributed to their development of a PPMD, two barrier categories, and an enabler category that influenced their help-seeking behaviors. Through navigation of formal and informal help, women were able to begin to reclaim the mothering instincts they had lost to mental illness.	19
Guy, Sterling, Walker and Harrison (2014) United States	To use Jorm’s (2000) framework to understand mental health literacy in one sample of lower income women to share participants’ knowledge and beliefs about recognizing postpartum depressive symptoms and seeking help for these symptoms.	25 women, 24.3 years (mean)	Postnatal depression, inclusion CES-D ** over 16	Prenatal care through Medicaid	Focus groups Deductive analysis based on mental health literacy conceptual categories (Jorm), qualitative thematic analysis	Women recognized behavioral changes indicating mental distress, but fears prevented them from seeking help, and some resorted to risky behaviors.	18
Holopainen (2002) Australia	To explore women’s experiences of support and treatment for postnatal depression.	7 women, age 24–43 years	Postnatal depression, inclusion current or recent perinatal depression	Postnatal support group in community health services, sexual assault center	Interviews Phenomenology (Creswell)	Women did not know where to seek help and were unaware of perinatal depression. Women were ambivalent of the use of medication. Women had ambivalent personal beliefs of being weak. Women wanted to be understood. Programs did not involve the family.	20
Jarrett (2015) United Kingdom	To explore women’s perspective of care from GPs and midwives, when they experience symptoms of depression during pregnancy.	22 women, age not known	Prenatal depression, inclusion self-reported symptoms of depression	Internet discussion group for mental health during pregnancy	Online questions in two discussion forums Qualitative descriptive design (Neergaard), thematic analysis (Braun and Clarke)	Themes were identified from the data including women’s disclosure of symptoms to GP’s and midwives; lack of knowledge of perinatal mental health among health providers; attitudes of staff and systemic issues as barriers to good care; anti-depressant therapy and care that women found helpful.	19
Jesse, Dolbier, and Blanchard (2008) United States	To identify: (1) potential barriers to sharing depressive symptoms with health care providers, (2) suggestions about how health care providers can best help women with depressive symptoms overcome barriers to seeking care, and (3) feedback regarding prenatal interventions that might be helpful for low-income women with depressive symptoms or depression in pregnancy.	21 women, all over 18 years	Prenatal depressive disorders, inclusion if assessed with high psychosocial risk in pregnancy	Prenatal clinic	Focus groups and two individual interviews Content analysis	Participants identified themes regarding barriers to seeking help. These were: (1) lack of trust, (2) judgment/stigma, (3) dissatisfaction with the health care system, and (4) not wanting help. Themes identified regarding overcoming barriers were: (1) facilitating trust and (2) offering support and help.	17
Letourneau, Duffett-Leger, Stewart, Hegadoren, Dennis et al. (2007) Canada	To assess the support needs, support resources, barriers to support, and preferences for support intervention for women with postpartum depression.	52 women, 31.3 years (mean)	Postnatal depression, inclusion depressive symptoms within past 2 years, 12 weeks of delivery, for longer than 2 weeks	Settings within integrated mental health services and postpartum follow-up	Interviews, group interviews Thematic content analysis	For most mothers, one-on-one support was preferred when postpartum depression is recognized. Group support should be available once the mothers start to feel better and are able to comfortably interact with other mothers in a group format.	15
McCarthy and McMahon (2008) New Zealand	To investigate the acceptance and experience of treatment for postnatal depression.	15 women, age 27–41 years	Postnatal depression, inclusion diagnosis of postnatal depression and in treatment since 3–12 months, with antidepressant medication	Community mental health setting	Interviews Grounded theory, “analytic induction method” (Glaser and Strauss)	The majority of women interviewed had reached “crisis point” before they sought and received treatment. The stigma attached to an inability to cope and being a “bad mother” emerged as the main barrier to seeking help earlier. In addition, women were unable to differentiate between “normal” levels of postpartum distress and depressive symptoms that might require intervention. Talking about their distress and experiences, both with health professionals and other mothers, was regarded as of primary importance in the recovery process.	16
Raymond, Pratt, Godecker, Harrisin, Kim et al. (2014) United States	To explore the following research objectives: What perceived needs do women describe they have in relation to their mental health through the perinatal period? What help do women describe current seeking in relation to addressing mental health concerns during the perinatal period? What support do women describe wanting for addressing mental health concerns during the perinatal period?	37 women, 27.5 years (average)	Perinatal mental health needs, inclusion if receiving prenatal or postnatal care	Three healthcare clinics in disadvantaged parts of urban areas	Focus groups Thematic analysis, social constructivist version of grounded theory (Charmaz)	Thirteen themes emerged which were described in relation to mental health needs, help currently accessed and the type of support wanted. The themes included the various mental health needs including dealing with changing moods, depression, feelings of isolation, worrying and a sense of being burdened. Women described using a limited range of supports and help. Participants expressed a preference for mental health support that was empowerment focused in its orientation, including peer support. Women also described the compounding effect that social and economic stresses had on their mental health.	19
Sword, Busser, Ganann, McMillan and Swinton (2008) Canada	To explore care seeking among women after public health nurse referral for probable postpartum depression, including responses to being referred, specific factors that hindered or facilitated care seeking, experiences seeking care, and responses to interventions offered.	18 women, 29.4 years (mean)	Postnatal depression, inclusion EPDS * 12	Public health setting with early prevention	Interviews Socioecological framework of health services, conventional content analysis (Hsieh)	Women’s normalizing of symptoms, limited understanding of postpartum depression, waiting for symptom improvement, discomfort discussing mental health concerns, and fears deterred care seeking; symptom awareness and not feeling like oneself were facilitating influences. Family and friends sometimes hindered care seeking because they, too, normalized symptoms or had limited understanding of postpartum depression. Care seeking was facilitated when women encouraged a health professional visit or expressed worry and concern.	18
Thomas, Scharp and Paxman (2014) United States	What IM ***-derived constructs permeate mothers’ talk about the postpartum depression experience?	30 women, age not known	Postnatal depression, inclusion if writing represents a woman’s 1st person account of her experiences	Online discussion group on postpartum depression	Anonymous online stories IM ***, inductive open coding; closed-coding procedure (Strauss and Corbin)	Five constructs (i.e., social norms; severity; barriers to help-seeking; facilitators to, and cues to action for, help-seeking; and self-efficacy) were prevalent.	18
Viveiros and Darling (2018) Canada	To explore access to PMH care services from a midwifery perspective: What do recipients of midwifery care perceive to be the factors that prevent or facilitate access to mental health care for women who experience depression, anxiety, and other mental health concerns in the perinatal period?	16 women, all over 18 years	Perinatal mental health, inclusion if self-identification of mental health concerns	Midwifery care	Interviews, focus groups Deductive thematic analysis (Braun and Clarke) based on Levesque’s et al. framework on access to health care	Five salient themes emerged from the data: cultural values, knowledge, relationships, flexibility, and system gaps. Barriers and facilitators to accessing perinatal mental health services are grouped under each theme. Stigma and fear, broken referral pathways, distant service location, lack of number/capacity of specialized services, baby-centeredness, discharge from midwifery care at six weeks postpartum, and cost were barriers to accessing care. Information and midwives’ knowledge/experience were context-specific factors that could hinder or facilitate access. Continuity, community, and advocacy were facilitators to accessing care.	19

* EPDS: Edinburgh Postnatal Depression Scale. ** CES-D: Center for Epidemiologic Study-Depression Scale. *** IM: Integrative Model of Behavioral Prediction (Fishbein).

## Data Availability

We have published the themes and preliminary clusters of the analysis of the meta-ethnography as Supplementary Table S1 and Figure S1. Please contact the authors for additional information.

## Supplementary Materials

The following are available online at https://www.mdpi.com/article/10.3390/ijerph18105226/s1, Table S1. All themes extracted, and Figure S1. Preliminary Clusters: (a) Cluster own feelings; (b) Cluster system; (c) Cluster taking action.
