# Supplementary material for: Contemplating Help-Seeking in Perinatal Psychological Distress—A Meta-Ethnography
Source: Int J Environ Res Public Health. 2021 May 14;18(10):5226. doi: 10.3390/ijerph18105226 (PMC8156805; doi:10.3390/ijerph18105226)
Supplement: Supplementary file 1 [file ijerph-18-05226-s001.zip › ijerph-1176642-supplementary.pdf]

Supplementary Materials

Table S1. All themes extracted.

Meaning units thematized
Ability-to-ask-to-approach-to-engage
Ambivalence-emotional-turmoil
Awareness-oneself-or-family
Being identified and helped routinely incorporating MH issues
Birth trauma
Blaming partner hindering access to care
Bodily-Sensation
Communication-style-of-HCP-as-causing-distress
Comparing with others
Comprehensive maternal-child care as enabler to seeking help including physical psychological and emotional needs
Conflicting views of medicine and possible side-effects both from women's and HCP's perspectives
Continuity-of-care An existing relationship with a HCP as a facilitator to the use of MHS
Costs too much related to services as a barrier
Crisis-dead end-ultimate-situation-before-seeking-help
Crying
Denial
Difficulties in obtaining reliable information as a barrier
Difficulties-breastfeeding-baby
Disclosure could mean kids in custody
Discontinuity of care as a barrier
Discussion-on-normalization
Dissatisfaction with health care as a barrier
Downward-spiral-PPMD
E-health options were also seen as facilitators by mothers
Exhaustion, fatigue, not energy to ask for help
Expectations-(Perfect)-Motherhood-vs-Not-the-perfect-in-norms-perspective
Extracts-from-data-experiences
Falling to pieces and anxiety with baby led to help-seeking
Fear of psychiatric care and being seen as mad
Feeling unworthy of support
Financial problems
Focusing on current difficulties rather than past difficulties - facilitator
For-Professionals-Message-to-note

**Meaning units thematized**
Friends unsupportive
Guilt-Shame-Embarassment-perhaps-also-silences-the-person-not-perfect
Having-to-take-medicine-as-barrier
HCP should consider the family as a whole not focus only on the baby - a facilitator
HCPs helpful when providing access to care, asking, understanding
Helplessness
Help-seeking-as-longer-time-prior-to-recovery
Holistic-Whole-to-support-dyad emotional-physical
Important to be listened to and being taken seriously and you are not the only one and there is hope for improvement
Inability to cope
Isolation-Loneliness-no-contacts-no-communication-no-trusting-relationships
Lack of knowledge on PMH and PPD from the woman's and HCPs' perspective
Lack of shared experiences, loneliness
Lacking continuity of care for follow up and difficult to access care
Lacking-support
Length of wait times for services as a barrier
Lived experience with beliefs causes barrier to seeking help
Lost control
Lost-Confusion-do-not-know-what-it-is-and-happens-do-not-know-one-has-depression
Matching-what-mother-needs-and-help
Media-Public-knowledge-forcing-to-behave
Medication risks not informed reliably or with side-effects
Medication-makes-you-function
Mental-Ill-Health-Forces-to-behave-naming-according-to-diagnosis
Minimizing the problem by HCPs and family caused delay
Mothers-own-background-causes-challenges
Needed proactive partner support
Needed services to access care
Needing-Compassion-and-Care-perhaps-without-success
Needing-encouragement_to-seek-help-and-be-engaged
No decision making process
Not easy to disclose to HCPs, confidence important and belief that there is help
Not-knowing-where-to-seek-help-or-how
Overwhelming-many-barriers-on-multiple-levels
Own-choices-made-when-possible
Panic

Meaning units thematized
Peer support in help seeking and subsequent care
Perceived health care professional shortage as a barrier
Proactive home services
Range of emotions
Referred-to-specialised-care
Relying mostly on oneself, barrier for seeking help
Seemingly rushed and uncaring health care professional can deter women from accessing services
Self-help-activities
Services available at home or in the neighbourhood facilitate use of services
Sleeping-unwell-and-so-much
Someone else close took control and asked for help
Spiritual-support-as-enabler
Stigma, Being labelled with a mental illness as a barrier to their access of mental health services
Stigma-of-mental-illness-makes-one-feel-worse-how-they-look-at-you-and-how-you-feel
The physical environment in which mental health care services are provided may act as barriers
they would not talk to their health professionals until 'the time is right'
Thinking about reasons
Time constraints and a focus on the baby both before after birth
Trustworthy readily available and culturally sensitive (building trust) health care professional facilitates the use of services
Trying hard to get help when reaching a point where suicidal ideation begun and thoughts on hurting the baby
Trying to look for alternatives to health care
Unable to care for the baby-or-causes-inadequacy-feelings
Unable to open up
Wants-help-but-cancels-appointments
Women did not know where to find help
Worry about how the family could go on

(a)

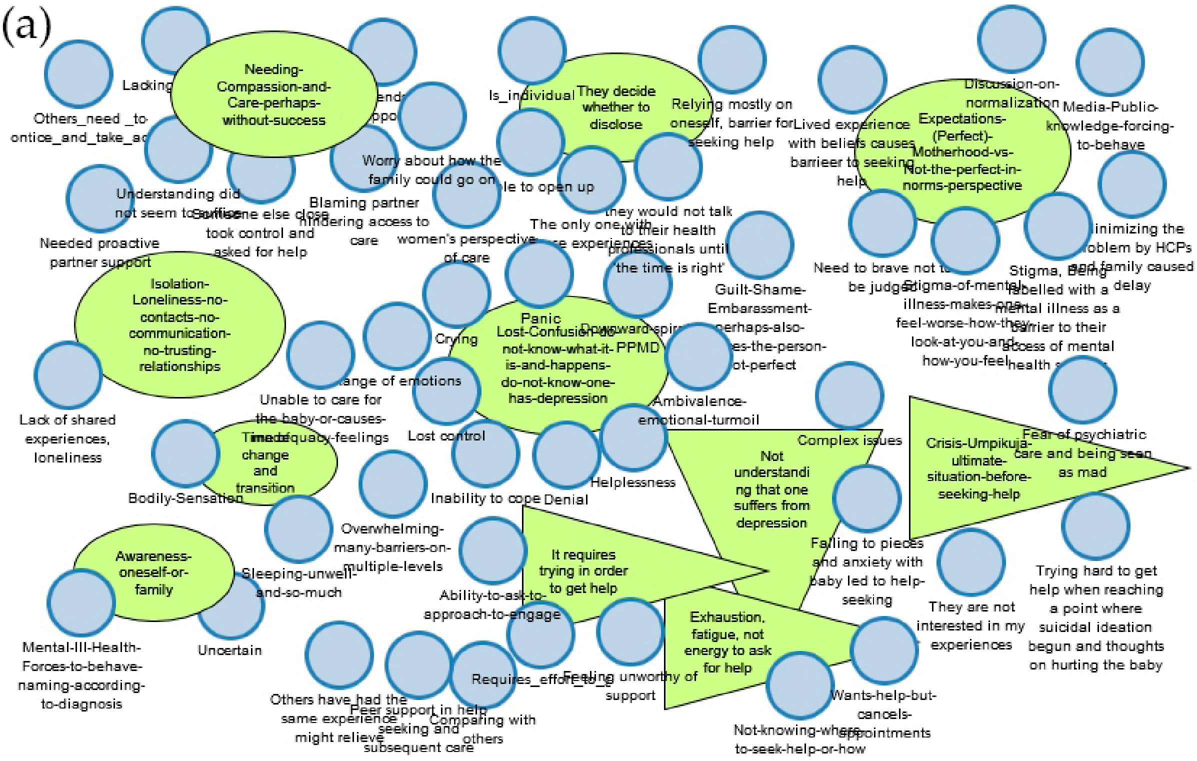

Needing-Compassion-and-Care-perhaps-without-success
Others_need _to-ontice_and_take_ac
They decide whether to disclose
Relying mostly on oneself, barrier for seeking help
Lived experience with beliefs causes barrieer to seeking help
Expectations-(Perfect)-Motherhood-vs-Not-the-perfect-in-norms-perspective
Media-Public-knowledge-forcing-to-behave
Worry about how the family could go on
Blaming partner hindering access to care
Needed proactive partner support
Isolation-Loneliness-no-contacts-no-communication-no-trusting-relationships
Lack of shared experiences, loneliness
Guilt-Shame-Embarassment-
Need to brave not to be judged
Stigma, Being labelled with a mental illness as a barrier to their access of mental health
Panic
Crying
Unable to care for the baby-or-causes-
Lost control
Ambivalence-emotional-turmoil
Complex issues
Not understandi ng that one suffers from depression
Crisis-Umpikuja-ultimate-situation-before-seeking-help
Fear of psychiatric care and being seen as mad
Bodily-Sensation
Inability to cope
Denial
Helplessness
Awareness-oneself-or-family
Overwhelming-many-barriers-on-multiple-levels
It requires trying in order to get help
Ability-to-ask-to-approach-to-engage
Falling to pieces and anxiety with baby led to help-seeking
They are not interested in my experiences
Trying hard to get help when reaching a point where suicidal ideation begun and thoughts on hurting the baby
Mental-Ill-Health-Forces-to-behave-naming-according-to-diagnosis
Uncertain
Exhaustion, fatigue, not energy to ask for help
Wants-help-but-cancels-appointments
Not-knowing-where-to-seek-help-or-how

(b)

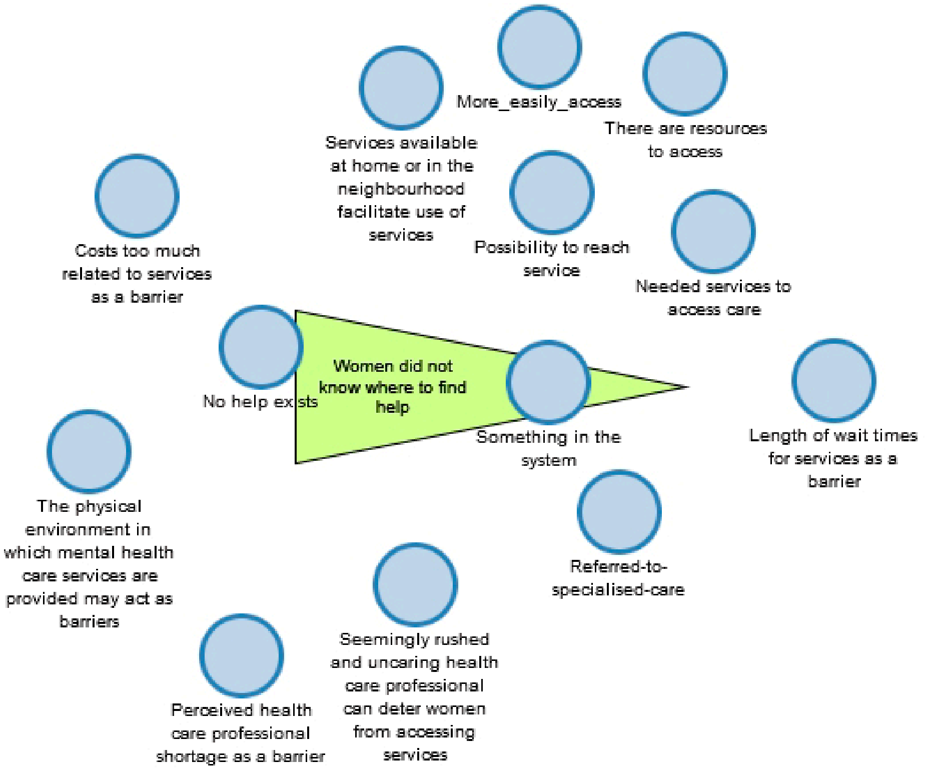

More_easily_access
Services available at home or in the neighbourhood facilitate use of services
There are resources to access
Costs too much related to services as a barrier
Possibility to reach service
Needed services to access care
No help exists
Women did not know where to find help
Something in the system
Length of wait times for services as a barrier
The physical environment in which mental health care services are provided may act as barriers
Referred-to-specialised-care
Seemingly rushed and uncaring health care professional can deter women from accessing services
Perceived health care professional shortage as a barrier

(c)

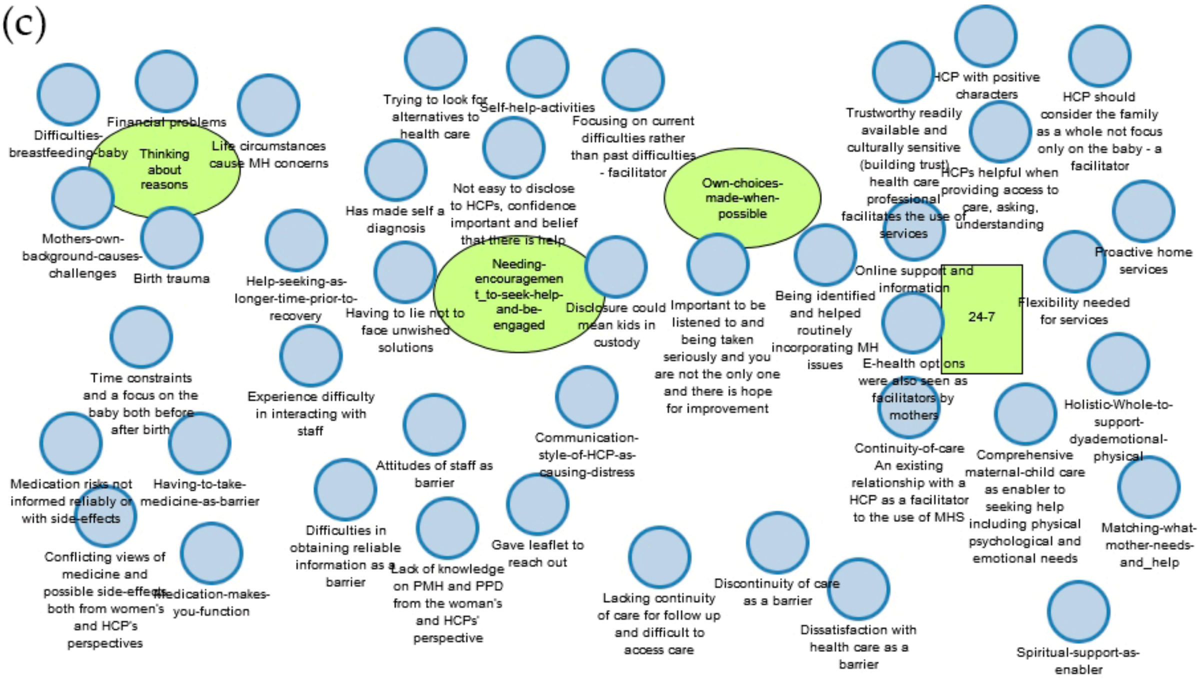

Figure S1. Preliminary Clusters: (**a**) Cluster own feelings; (**b**) Cluster system; (**c**) Cluster taking action.
